# Evaluating the Tubridge™ flow diverter for large cavernous carotid artery aneurysms

**DOI:** 10.1186/s41016-020-00215-z

**Published:** 2020-12-01

**Authors:** Luqiong Jia, Jiejun Wang, Longhui Zhang, Yunfeng Zhang, Wei You, Xinjian Yang, Ming Lv

**Affiliations:** 1 Department of Interventional Neuroradiology, Beijing Neurosurgical Institute and Beijing Tian Tan Hospital, Capital Medical University, Beijing, 100070 China; 2 Department of Imaging and Nuclear Medicine, Baoding No. 1 Central Hospital, Baoding, Hebei China

**Keywords:** Tubridge, Flow diverter, Aneurysm, Large, Cavernous

## Abstract

**Background:**

The Tubridge™ flow diverter (TFD) was recently developed in China; however, its safety and efficacy in treating large cavernous carotid artery aneurysms (LCCAs) are unclear. Our objective was to evaluate the safety and efficacy of the TFD in patients receiving TFDs to treat LCCAs (10–25 mm).

**Methods:**

Between June 2013 and May 2014, seven patients with LCCAs were enrolled in our study, and all seven patients underwent TFD implantation combined with coils.

**Results:**

Angiographic follow-up images were available for all seven patients at a median of 57.5 ± 16.7 (range, 6–69) months. Seven patients obtained favorable angiographic results defined as O’Kelly–Marotta Scale C and D. Clinical follow-up data were available for all seven patients at a median of 73.32 ± 3.6 (range, 66–78) months. No patients developed new neurological deficits. Six patients achieved a modified Rankin scale score of 0, and diplopia improved in the remaining patient.

**Conclusions:**

The results were excellent for the aneurysms treated with TFDs in our patients with LCCAs. TFDs are feasible for the treatment of LCCAs, but a multicenter, controlled clinical trial is needed to evaluate the long-term safety and efficacy of the TFD to treat LCCAs.

## Background

Cavernous carotid artery aneurysms (CCAs) account for ≤ 5% of all intracranial aneurysms [1–5]. According to the etiology, CAAs can be divided into traumatic, mycotic, and idiopathic, and idiopathic aneurysms are the most common. Because CCAs are located in the extradural space, symptomatic and large aneurysms usually manifest as symptoms and signs of a mass effect on the surrounding structures [3, 6, 7], and patients present with intractable cranial neuropathy requiring intervention [8]. The treatment of large intracranial aneurysms, compared with small aneurysms, is associated with high complication and recurrence rates [9, 10].

Several flow-diverting devices have been developed with the goal of changing the intrasaccular hemodynamics and reconstructing the parent artery, namely, the Pipeline flow diverter (Covidien, Irvine, CA), the Flow-Redirection Endoluminal Device (FRED; MicroVention, Tustin, CA), the Silk flow diverter (Balt Extrusion, Montmorency, France), and the Surpass stent (Stryker Neurovascular, Kalamazoo, MI). The frequency of use of these devices has increased sharply in the treatment of intracranial aneurysms. The Tubridge™ flow diverter (TFD) is a braided, self-expanding device with flared ends. Compared with other flow diverter devices, TFDs are made of a nickel–titanium alloy, which has the advantages of super-elasticity and shape-holding memory. In addition, the use of platinum–iridium radiopaque microfilaments allows for improved visualization of the length and diameter during the endovascular procedure. TFDs are available in several lengths (12–45 mm) and diameters (2.5–6.5 mm) and can provide a high degree of metal coverage (approximately 30.0–35.0%) at the aneurysmal neck after full opening, with a lower shortening rate [11].

Previously, a multicenter, prospective, randomized, controlled clinical trial verified the safety and efficacy of the TFD in unruptured large and giant intracranial aneurysms [12]. However, as a novel device, outcomes of TFDs to treat large CCAs (LCCAs, 10–25 mm) have not yet been clarified. The purpose of this study was to evaluate the safety and efficacy of TFDs in the treatment of LCCAs.

## Methods

### Inclusion and exclusion criteria

The inclusion criteria are the patient should be between 18 and 75 years of age, with a cavernous sinus aneurysm with a diameter of 10 mm or greater and a neck of 4 mm or greater, and the patient agreed to be treated with TFD.

The exclusion criteria are pregnant woman, ruptured aneurysm, and patients have other cerebrovascular diseases such as arteriovenous malformations or arteriovenous fistulas.

All patients were from Beijing Tiantan Hospital.

### Patient population

This was a single-center, retrospective study that was approved by the institutional Ethics Committee. Written informed consent for study inclusion was obtained from all patients. Between June 2013 and May 2014, 1378 patients came to Beijing Tiantan Hospital for endovascular treatment of intracranial aneurysm. A total of 132 patients were diagnosed with CCAs. Among the 132 patients, 67 patients were diagnosed with LCCAs, and seven patients received TFDs to treat LCCAs.

### Endovascular procedure

For all enrolled patients, dual antiplatelet therapy (300 mg/day acetylsalicylic acid (ASA) and 75 mg/day clopidogrel) was given for at least 3 days before the endovascular procedure. All TFD placement procedures were performed under general anesthesia and via the transfemoral approach. Using the preoperative road map, a Traxcess-14 (Micro-Vention, Tustin, CA) microguidewire carried the Endopipe (Microport, Shanghai, China) stent catheter to the middle cerebral artery, and then an appropriate microcatheter was carried by the Traxcess-14 microguidewire and navigated into the aneurysmal sac. Next, we withdrew the microguidewire and performed additional coiling in all the aneurysms through the microcatheter to the aneurysmal sac. Then, we delivered the appropriate TFD through the Endopipe stent catheter and released the TFD after satisfactory positioning. The treatment procedure was well-documented.

### Postoperative medication

Each patient was prescribed 300 mg of ASA plus 75 mg of clopidogrel for 6 weeks, then the dose of ASA was reduced to 100 mg from 6 weeks to 3 months. Clopidogrel was discontinued after 3 months, and 100 mg of ASA was continued indefinitely.

### Imaging and clinical assessment

We used the O’Kelly–Marotta Scale [13] to classify both the immediate postoperative angiographic results and the follow-up angiographic results. This grading scale is used to evaluate aneurysms treated with flow diversion and indicates both the degree of contrast stasis and the amount of aneurysm filling. The scale is widely used to evaluate the efficacy of flow diverter devices such as the Pipeline and Silk devices. We defined O’Kelly–Marotta Scale C or D as a favorable outcome. Angiographic results were confirmed by at least two experienced neurointerventionists. We collected each patient’s clinical information, including whether the original symptom had improved and whether any new symptoms appeared, postprocedure.

## Results

### Patient and aneurysm characteristics

Between June 2013 and May 2014, seven patients each with a large cavernous CCA were enrolled in our study. Clinical presentation included diplopia in four patients (one accompanied by blepharoptosis), ocular pain in one patient, facial tic in one patient, and right frontal sinus pain in one patient (Table 1). No patients had a history of subarachnoid hemorrhage or other vascular genetic histories such as arteriovenous malformation. Table 1 shows the patients’ demographics and clinical information.

**Table 1:** Patients’ demographics and clinical information

Case	Symptoms	Size (mm)/side of aneurysm	Size of TFD (mm)	O’Kelly–Marotta Scale
1	Diplopia and blepharoptosis	11.1 × 9.5/L*	4.0 × 45	B3
2	Ocular pain	20.9 × 10.1/R*	6.0 × 35	A3
3	Facial tic	12.1 × 11.5/R	4.5 × 30	B3
4	Diplopia	19.0 × 13.9/L	4.5 × 25	C3
5	Frontal pain	20.4 × 16.2/R	5.5 × 45	B3
6	Diplopia	21.0 × 16.3/R	4.5 × 45	A3
7	Diplopia	23.6 × 22.5/L	4.5 × 35	B3

*L* Left, *R* Right

### Immediate angiographic and clinical results

We implanted seven TFDs; each patient was treated with a single TFD plus coils. Six of the seven patients received loose packing of the aneurysmal sac, and only patient 4 received dense packing of the aneurysmal sac. Two patients were graded as O’Kelly–Marotta Scale grade A, and four patients were graded as B; one patient was graded as C (Table 1). No new neurological deficits developed after the endovascular treatment in any of the patients, and no bleeding or ischemic events occurred during or after the endovascular treatment.

### Angiographic follow-up results

**Fig. 1:**
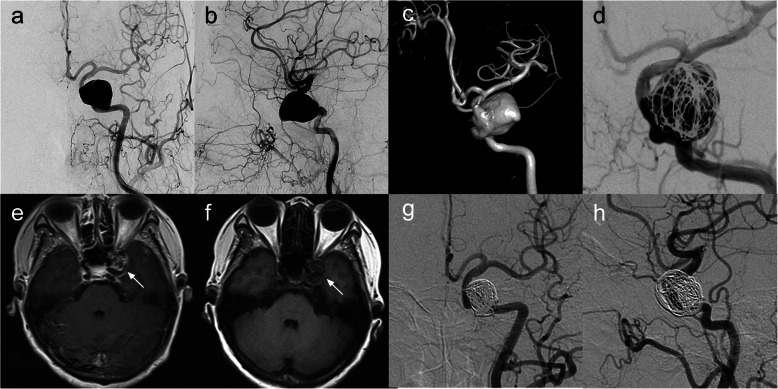
Images from a patient with a left large cavernous carotid artery aneurysm (patient 1). **a**–**c** Preoperative angiograms of the left internal carotid artery showing a large cavernous carotid artery aneurysm. **d** Immediately postoperative angiogram of the left internal artery showing reconstruction of the parent vessel and contrast stasis in the lumen of the aneurysm. MRI 4 months posttreatment (**f**) compared with the 2-day posttreatment MRI (**e**) showing a slight reduction of the aneurysm size (white arrow) and increased space around the brainstem. **g**, **h** Angiogram 66 months posttreatment showing the occluded aneurysm and reconstruction of the left internal carotid artery. MRI, magnetic resonance image

**Fig. 2:**
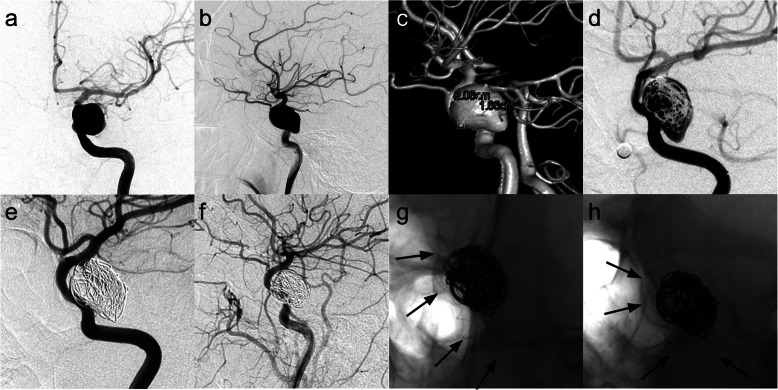
Images from a patient with a left large cavernous carotid artery aneurysm (patient 1). **a**–**c** Preoperative angiograms of the left internal carotid artery showing a large cavernous carotid artery aneurysm measuring 20.6 × 16.6 mm. **d** Immediately postoperative angiogram of the left internal artery showing reconstruction of the parent vessel and contrast stasis in the lumen of the aneurysm. **e**, **f** Angiograms 66 months posttreatment showing the occluded aneurysm and reconstruction of the left internal carotid artery. **g**, **h** Unsubtracted view of 66 months posttreatment confirming that the stent was in good shape with no compression

**Fig. 3:**
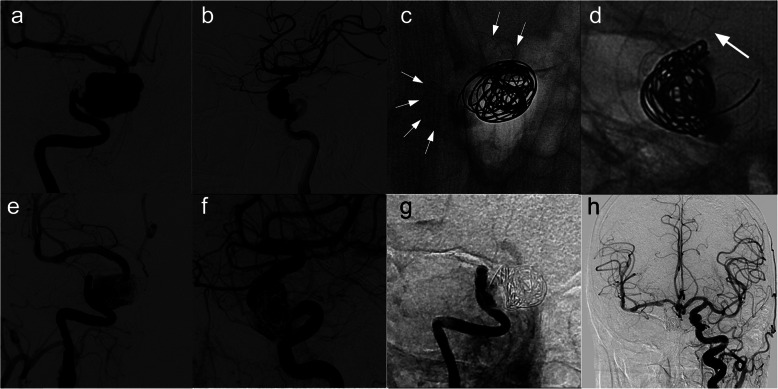
Images from a patient with a right large cavernous carotid artery aneurysm (patient 6). **a**, **b** Preoperative angiograms of the right internal carotid artery showing a large cavernous carotid artery aneurysm. **c**, **d** Intraprocedural unsubtracted view showing successful insertion of the TFD (4.5 × 45 mm). **e**, **f** Immediately postoperative angiogram of the right internal carotid artery showing reconstruction of the parent vessel and contrast stasis in the lumen of the aneurysm. **g** Angiogram 6 months posttreatment showing occlusion of the right internal carotid artery. **h** Angiogram of the left internal carotid artery confirming that the anterior communicating artery provided sufficient blood for the right anterior circulation.

We selected the final digital subtraction angiographic follow-up image for each patient as the time point to evaluate the efficacy of TFD placement. Angiographic follow-up data were obtained for all seven patients (Table 2), with a median imaging follow-up period of 57.5 ± 16.7 (range, 6–69) months. All seven patients obtained favorable angiographic follow-up results (five patients’ O’Kelly–Marotta Scale grades were D (Figs. 1 and 2), and two patients were grade C). In one of the seven patients, (Fig. 3), parent artery occlusion was seen in the 6-month digital subtraction angiographic image. The occlusion was located in the TFD, but there was no clinical manifestation associated with cerebral infarction because the left internal carotid artery provided sufficient blood for the right anterior circulation through the anterior communicating artery.

**Table 2:** Angiographic and clinical follow-up data

Case	Angiographic follow-ups				Clinical follow-ups	
	Time, months	Method	O’Kelly–Marotta Scale	Parent artery	Time, months	mRS* score
1	66	DSA*	D	Patent	73	0
2	62	DSA	D	Patent	69	0
3	69	DSA	D	Patent	74	0
4	51	DSA	C	Patent	66	1
5	17	DSA	C	Patent	78	0
6	6	DSA	D	Occlusion	74	0
7	6	DSA	D	Patent	71	0

*DSA* Digital subtraction angiography, *mRs* Modified Rankin scale

### Clinical outcomes

Clinical follow-up data were available for all seven patients at a median of 73.32 ± 3.6 (range, 66–78) months. No new neurological deficits were observed in any patient. Six patients achieved a modified Rankin scale score of 0, and the remaining patient experienced improved diplopia (Table 2).

## Discussion

Compared with small aneurysms, treating large aneurysms is technically challenging, with a much higher complication and recanalization rate [8, 14]. Long-term angiographic outcomes showed that recurrence rates for large aneurysms treated with coiling alone or stent-assisted coiling were 57.9% and 23.5%, respectively [15], indicating that satisfactory outcomes cannot be achieved via conventional endovascular treatment. Parent artery occlusion can be used to treat large aneurysms, but this requires a negative balloon occlusion test, and new aneurysms occurred in other areas in 4.5% of patients after parent artery occlusion [16]. In addition, when treating LCCAs, our goals are to reduce the risk of rupture and thromboembolism and relieve cerebral nerve paralysis caused by the aneurysmal mass effect. Recently, higher numbers of large aneurysms are being treated with flow diverter devices, and the efficacy and safety of these devices are being proven. Flow diverter devices contrast with the traditional treatment concept of intracranial aneurysmal sac tamping and reconstruct the parental artery, which is a big step in the treatment of intracranial aneurysms (in Table 3, we compared the advantages and disadvantages of the three endovascular treatment modalities). In the present study, we reported our preliminary findings related to the use of TFDs in LCCAs.

**Table 3:** Advantages and disadvantages of the three endovascular treatment modalities

Treatment modalities	Advantages	Disadvantages
Coils	1. Do not need to take antiplatelet drugs after endovascular treatment.	1. Wide-necked aneurysm is not applicable. 2. There is a risk of coil escape and a relatively high recurrence rate. 3. It needs to be operated in the lumen of the aneurysm, and there is a risk of aneurysm rupture.
Stent-assisted coils	1. The incidence of escape risk of coils is relatively low.	1. Requires platelet inhibition, which can lead to long-term complications. 2. A relatively high recurrence rate. 3. It needs to be operated in the lumen of the aneurysm, and there is a risk of aneurysm rupture.
Flow diverter device	1. Higher embolization rate. 2. No operation is performed in the lumen of the aneurysm, reducing the risk of intraoperative aneurysm rupture.	1. Branch occlusion or perforating branch occlusion is likely to occur. 2. Requires platelet inhibition, which can lead to long-term complications.

In our series, angiographic follow-up data were obtained for all seven patients (Table 2) with a median imaging follow-up period of 57.5 ± 16.7 (range, 6–69) months. All seven patients obtained favorable angiographic follow-up results. Lin et al. reported that complete aneurysm occlusion was achieved in a higher proportion of the pipeline plus coils compared with Pipeline only (93.1% vs 74.7%, *P* = 0.03 ) [17]. O’Kelly et al. reported that for patients presenting with cranial nerve deficits (18 cavernous aneurysms), 11 patients experienced resolution (61%) [18]. In our clinical follow-up, patients achieved even better results, with six patients experiencing complete resolution (85.7%) of the aneurysmal mass effect symptoms.

The use of flow diverter devices theoretically does not require coiling. However, for large, complex aneurysms, additional coils could play a role in improving occlusion rates and decreasing the risk of catastrophic aneurysm rupture after the use of flow diverter stents [17, 19]. In our study, every patient was treated with a TFD and coils because we believe that the additional coils accelerate thrombus formation to decrease the pressure from the aneurysmal sac caused by blood retention within the sac after TFD implantation. Jing et al. [20] have also reported that adjunctive coiling with the TFD can reduce intra-aneurysmal flow velocity and wall shear stress, promoting thrombosis formation and embolization of aneurysms. In an earlier experience using the Pipeline flow diverter, Siddiqui et al. [21] recommended avoiding dense packing of the aneurysmal sac because this can lead to acute thrombotic or compressive occlusion. Our findings were similar; six patients were treated with low coil-packing densities, and their cranial nerve deficits resolved completely. The only patient (patient 4) treated with dense aneurysmal packing obtained subtotal cranial nerve deficit improvement. Although we observed good outcomes for loose embolized aneurysms, because only one patient received dense aneurysmal packing, we still need a larger number of records to reach the conclusion of loose aneurysmal packing does not affect alleviation of the mass effect.

The reported complication rates for ischemia and bleeding following aneurysmal repair are 5.5–9.76% and 2.0–6.1%, respectively, with morbidity and mortality rates of 9.8–17.7% and 3.8–4.9%, respectively [22, 23]; the incidence of complications is higher for giant aneurysms [24]. The incidence of complications in the flow diverter device group in our study was lower than that in conventional parent artery occlusion with a single coil and stent-assisted coiling when treating cavernous aneurysms [25]. In our case serious, one patient had occlusion of the parent artery but showed no signs of ischemia because the left internal carotid artery provided sufficient blood flow for the right anterior circulation through the anterior communicating artery. But the result needs to be taken seriously; not all patients can compensate adequately after unilateral internal carotid artery occlusion, and ischemic events may occur once compensation is insufficient. One meta-analysis indicated ischemic rate after flow diverter implantation was 7.5% [26], and a 9–10% incidence of ischemic events should be anticipated when using flow diverters for large aneurysms [12]. We did not encounter hemorrhagic complications, and the morbidity and mortality rates were both 0%.

Up to now, there has been no single report on the therapeutic effect of TFD for LCCAs, in a clinical trial of Liu et al [12], 37 patients with paraclinoid or cavernous aneurysms (aneurysm size 21.8, 7.5, 10.0–45 mm) were included, with a 6-month complete embolization rate of 75.7%. The article reported 1-year mortality rate of 4.88%, hemorrhagic stroke and ischemic stroke related to target vessel were 6.1% and 9.76%, respectively (these calculations include aneurysms of all sites and was not a simple cavernous segment aneurysm). But no long-term follow-up results were reported.

### Limitations

The present study involved only seven patients, because LCCAs are rare. In addition, not all patients underwent angiographic magnetic resonance imaging, so we were able to evaluate the resolution of patients’ mass effects only according to the resolution of their clinical symptoms; we had no clear imaging evidence. A prospective, multicenter, controlled clinical investigation with a large sample and long-term follow-up is essential.

## Conclusions

Our patients with LCCAs treated with TFDs obtained excellent results, with a high percentage of patients experiencing remission of their aneurysmal mass effect symptoms. TFDs could be feasible for treating LCCAs; however, a multicenter, randomized, controlled clinical trial with long-term follow-up is still necessary.

## Data Availability

Please contact the author for data requests.
